# Design and Application of Joule Heating Processes for Decarbonized Chemical and Advanced Material Synthesis

**DOI:** 10.1021/acs.iecr.4c02460

**Published:** 2024-11-04

**Authors:** Anthony Griffin, Mark Robertson, Zoe Gunter, Amy Coronado, Yizhi Xiang, Zhe Qiang

**Affiliations:** † School of Polymer Science and Engineering, The University of Southern Mississippi, Hattiesburg, Mississippi 39406, United States; ‡ Dave C. Swalm School of Chemical Engineering, Mississippi State University, Mississippi State, Mississippi 39762, United States

## Abstract

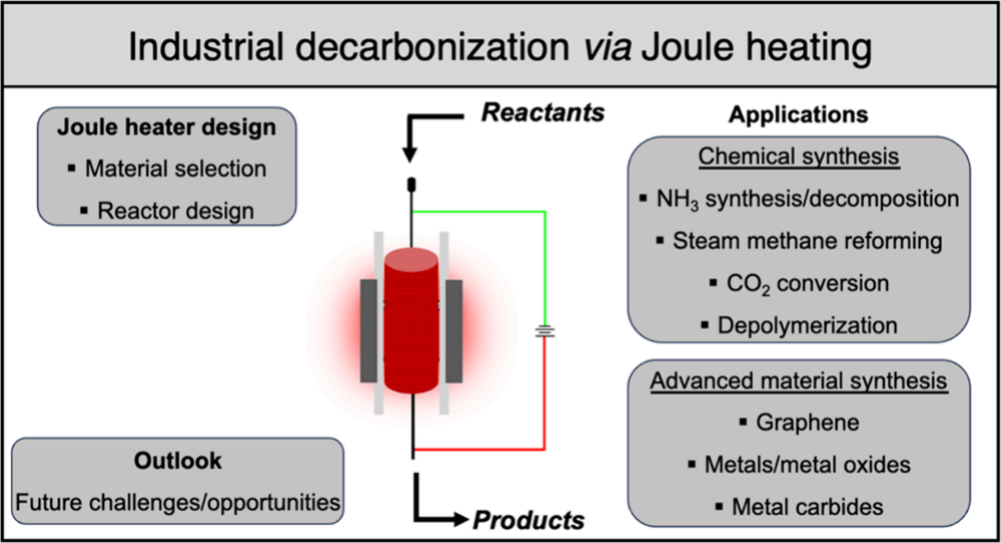

Atmospheric CO_2_ concentrations keep increasing at intensifying rates due to rising energy and material demands. The chemical production industry is a large energy consumer, responsible for up to 935 Mt of CO_2_ emissions per year, and decarbonization is its major goal moving forward. One of the primary sources of energy consumption and CO_2_ emissions in the chemical sector is associated with the production and use of heat for material synthesis, which conventionally was generated through the combustion of fossil fuels. To address this grand challenge, Joule heating has emerged as an alternative heating method that greatly increases process efficiency, reducing both energy consumption and greenhouse gas emissions. In this Review, we discuss the key concepts that govern these Joule heating processes including material selection and reactor design, as well as the current state-of-the-art in the literature for employing these processes to synthesize commodity chemicals along with advanced materials such as graphene, metal species, and metal carbides. Finally, we provide a perspective on future research avenues within this field, which can facilitate the widespread adoption of Joule heating for decarbonizing industrial processes.

## Introduction

CO_2_ has been released into the atmosphere at continuously increasing rates since the Industrial Revolution in large part due to anthropomorphic emissions as a result of increased energy demand, chemical production, and some agricultural practices. Together, these activities have resulted in a 50% increase in the atmospheric CO_2_ concentration within the past few centuries.^1,2^ Specifically, the continued rise of greenhouse gas (GHG) emissions is associated with a variety of adverse effects, including extreme weather patterns, disruptions in food supply, higher average global temperatures, and, if left unaddressed, a threat of mass extinction.^3−5^ The Sixth Assessment Report by the Intergovernmental Panel on Climate Change (IPCC) has outlined key Sustainable Development Goals focused on improvements to curtail the global threat of GHG emissions.^6^ One of these goals is focused on the development of innovation and sustainable practices to enable decarbonized industrial processes; here “decarbonized” refers to reducing the concentration of CO_2_ and other GHG gases in the atmosphere. The industrial sector accounted for 30% of all U.S. energy-related CO_2_ emissions in 2020 and the global chemical industry represents 5% of emissions worldwide.^7,8^ Noteworthily, the production of many commodity chemicals, including ammonia, olefins, and methanol, requires several energy-intensive reactions that involve relatively high reaction temperatures (700–1000 °C).^9^ As a result, the CO_2_ emissions from chemical production alone totaled 2 billion metric tons in 2022.^10^ As global chemical production is anticipated to quadruple by 2060,^11^ the feedstocks and processes for chemical synthesis must be improved to reduce the environmental impact of these industries in the coming decades. More specifically, a net zero emission scenario developed by the International Energy Agency (IEA) necessitates an 18% reduction of CO_2_ emissions within the chemical sector by 2030 (compared to 2022), intending to achieve net zero emissions by the year 2050.^10^ Additionally, the “Industrial Decarbonization Roadmap” outlined by the U.S. Department of Energy (DOE) highlighted four critical strategies for decarbonization, including developing and utilizing low-carbon fuels/feedstocks/energy sources, carbon capture/utilization/storage, industrial electrification, and increasing energy efficiency.^12^ Therefore, as the demand for chemicals is projected to continue steadily increasing,^8^ there is a strong need for technological innovation and breakthroughs to enable low carbon emission manufacturing processes. Decarbonizing the chemical industry is a complex challenge that will require a unified approach by integrating various strategies.^9,13,14^ These include carbon capture and storage (CCS),^15−18^ the use of biobased and renewable feedstocks,^19−23^ waste reduction and recycling to promote a circular economy,^24−28^ reduction in chemical demand,^9,29,30^ and the development of technologies to reduce the energy consumption of existing industrial processes.^13,31−34^

Approximately 80% of the energy consumed in the U.S. chemical industry is related to heat generation, which is most commonly through the combustion of fossil fuels.^14,35^ In these processes, thermal energy is required to preheat the reactants and maintain the reactor to desired temperatures in order to produce chemicals and materials at scale. Specifically, for the highly endothermic reaction processes, an array of up to 100 tubular catalytic reactors is positioned within a gas burner furnace for optimal heat distribution within the catalyst bed. This external heating/burning process through the combustion of fossil fuels leads to several sustainability challenges: 1) Heating across the volume of the reactor/catalyst-bed in many cases is not uniform, the steep temperature gradients can lead to side/undesired products or reduced conversion efficiency. 2) Start-up and shut-down times for these reactors can be very lengthy due to their large volumes, resulting in further energy consumption, fossil fuel combustion, and GHG emissions. 3) Insufficient heat flux requires the generation of temperatures much greater than that of the desired reaction to ensure sufficient heat transfer throughout the entirety of the reactor volume, ultimately leading to large amounts of heat waste.

There are several promising alternative methods to conventional gas fired systems that utilize electrified heating processes, including Joule,^36−38^ induction,^37−41^ plasma,^42−47^ and microwave heating.^48−55^ We note that this review article will be focusing on the Joule heating effect. These electrified heating strategies are not only more efficient than conventional fired systems, but are also capable of being integrated with renewable energy resources to further promote decarbonization efforts.^35^ Briefly, microwave heating involves the use of dielectric materials that absorb microwave radiation and convert the incident radiation to thermal energy.^48,49^ This strategy requires the use of insulating and reflecting materials to optimize microwave heating as well as careful consideration of microwave frequencies. An advantage of this method is that it is a noncontact heating approach that directly transfers energy to the target, though the use of microwaves requires additional design criteria as the heating elements must have the necessary interactions with microwaves and reactor materials must allow for microwave transmission. Induction heating involves the indirect transfer of electromagnetic energy to thermal energy, where an alternating electromagnetic field is generated with an induction coil which results in the generation of eddy currents within ferromagnetic objects.^56^ Similar to microwave heating, this is a noncontact process, while it requires the presence of ferromagnetic materials within the reactor. In addition to not requiring direct contact with the materials, using microwave and induction-based heating also enables the use of noncontinuous conductive materials to generate heat. More specifically, conductive particles can be subjected to generate heat locally, rather than requiring physical continuity between the heated material and the power source which can make the system more complex. The interested reader is directed to multiple other reviews to learn more about these processes.^37,48,49,56^

Joule heating, also known as ohmic or resistive heating, is a process that generates thermal energy directly in a material when an electric current passes through it. This occurs because the voltage difference between two points of the conductor creates a local electric field, which then accelerates charge carriers along the field direction and grants them kinetic energy. As these charged particles collide with the conductor’s ions, they can cause random motions and increased atomic vibrations, producing thermal energy to increase material temperature. Reactors utilizing Joule heating as a heat source can achieve increased heating efficiencies compared to the counterparts by combustion heating.^57^ Specifically, Joule heating allows direct heating of the reactor wall or elements where reactions occur, leading to a high uniformity of the heating process and low heat waste. Additionally, the Joule heating process is able to reach high reaction temperatures within seconds.^58^ This is in sharp contrast to long startup times of combustion-heated systems, providing further advantages to reduce energy consumption. Furthermore, the increased efficiencies of Joule heating reactions allow for the use of reactors that are much smaller than conventional ones, which can facilitate the intensification of various chemical and materials synthesis processes.^59^

Recently, there have been several examples of the electrified heating of industrial chemical processes, including the development of an experimental electrically heated steam cracker from Shell and Dow,^60^ as well as the use of electrically heated furnaces in an existing steam cracker plant by BASF, Linde, and SABIC.^61^ It is also worth noting that leveraging Joule heating for industrial reactions and processes provides a direct pathway to integrate renewable energy sources by converting the associated electrical energy to thermal energy.^35^ It is expected that renewable energy sources like solar, wind, and geothermal energy, will provide ∼70% of global electricity by 2050.^62^ By converting conventional heating processes to electrified heating, these renewable sources can be directly tapped for generating industrial process heat and even further reduce the associated GHG emissions of the heating processes. The potential to integrate with renewable energy sources, combined with size reduction in reactor design, also leads to more compact and efficient production setups while enabling delocalized and democratized chemical and energy synthesis. Smaller, more efficient reactors can be deployed closer to the point of use, reducing the need for large, centralized facilities and extensive distribution networks. Distributed production can enhance energy security, reduce transportation costs and emissions, and make advanced chemical and energy synthesis technologies more accessible to a wider range of industries and communities. Additionally, life cycle assessments and technoeconomic analyses have demonstrated reduced environmental impacts through the use of Joule heating compared to conventional processes in various syntheses, ranging from advanced materials to small molecules.^63−65^ Overall, this approach also allows for greater flexibility in meeting local demand and integrating with renewable energy sources, further contributing to sustainable and resilient energy and chemical production systems.

While many reviews explore the opportunities and barriers in decarbonizing the chemical industry or electrifying specific chemical reactions,^9,13,14,33^ this review primarily focuses on the core design concepts of Joule heating processes. Specifically, it emphasizes the selection of materials for Joule heating, reactor design, and the impact of these factors on various synthetic processes. Following discussion of Joule-heating reactor design and production, examples of chemical and advanced materials synthesis will be discussed in the two subsequent sections. Specifically, the electrification of several mature industrial chemical syntheses through the Joule heating effect will be covered, including ammonia synthesis/decomposition, CO_2_ reduction/conversion, steam methane reforming (SMR), and several others. Moreover, advanced materials syntheses, such as graphene, metals and metal oxides, metal carbides and other materials, using the Joule heating approach will be highlighted. In doing so, this work aims to provide a detailed overview of reactor design criteria for chemical and advanced materials synthesis to guide further Joule-heated reactor development. We also provide a brief perspective on the outlook of Joule heated processes for decarbonized syntheses, with consideration of future challenges and opportunities within this promising research area.

## Joule Heater Production and Performance

### Material Selection

**Figure 1:**
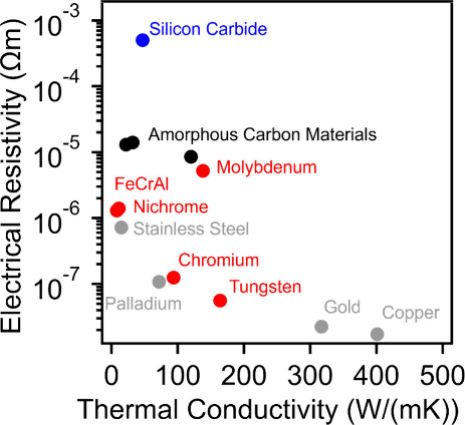
Comparison of electrical resistivities and thermal conductivities of materials commonly employed for Joule heating processes. The common materials are depicted by gray markers, metals commonly used for Joule heated processes are shown in red, and silicon carbide and carbon are presented in blue and black, respectively. The values presented in the figure are from refs (66−73).

Joule heating is the process of converting electrical energy to thermal energy as current passes through a conductor, described by Joule’s law as given in eq 1:
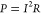
1where *P* is the power in Watts generated as a result of the flowing current, representing the amount of heat which can be produced per second. *I* is the electric current passing through the conductor in amperes, and *R* is the resistance of the conductor in ohms. The resistance of a material, or its opposition to the flow of electrons, results in the loss of energy from the flowing current through the generation of heat. The heat generation from the Joule heating process depends on the voltage and, consequently, the current applied. It is worth noting that, in practice, electrified heating is limited by maximum voltage and current constraints. Incorporating a resistive element with lower electrical resistance into a power source will result in drawing more current which could pose risks to the electrical components among other safety hazards. This highlights an advantage of using materials with higher electrical resistances as they can enable the use of lower voltage sources while maintaining smaller, more manageable currents. While the electrical resistivity of the material is a crucial parameter that can be adjusted to control the amount of heat generated for a given current, several other important properties must also be considered. Specifically, the thermal conductivity of the material used to generate Joule heat can determine the uniformity of heating throughout the Joule heated reactor, and materials with higher thermal conductivities are generally favored. It is worth noting that higher thermal conductivities are also associated with more rapid heat dissipation, but this can be largely mitigated through proper insulation techniques. Figure 1 illustrates the electrical resistivities and thermal conductivities of materials that have been used for Joule heating processes in addition to a few common materials to provide the reader with additional context.

Generally, metals exhibit higher thermal conductivities compared to ceramics and carbon materials, enabling their heating elements to be heated more uniformly. However, metals often have low electrical resistivities, which can necessitate increased power inputs to reach the desired reaction temperatures. Currently, the most commonly employed metals for Joule heating applications are nickel/chromium (Nichrome) and iron/chromium/aluminum (FeCrAl) alloys. Both of these materials are commercially available and can be shaped into various geometries to suit different applications. As demonstrated in Figure 1, Nichrome and FeCrAl exhibit electrical resistivities that are orders of magnitude higher than common metals like gold, copper, and palladium, enabling their use as materials for Joule heated reactors.^66−73^ Chromium, tungsten, and molybdenum have also been employed as Joule heating materials for various processes in the literature.^74,75^ However, they generally exhibit lower efficiencies compared to Nichrome and FeCrAl derived materials, while their high cost makes them less suitable for large production. Generally, the maximum operating temperature of these materials corresponds to the melting points of the metals which are typically between 1000 °C – 2000 °C, above which, the metals would experience catastrophic failure. However, it is important to note an additional thermal transition which may affect the performance of metal-derived systems, the Tammann temperature. The Tammann temperature of metals is typically approximated as half the melting temperature, on an absolute scale, and is characterized by a sudden increase in atomic mobility. Operating at temperatures above the Tammann temperature could result in morphological changes of the metal species, potentially resulting in changes in electrical and thermal properties, in addition to sintering together of metal components. The majority of these metals can be susceptible to oxidation by various chemical processes and often require coatings to prevent their degradation during processing or inert atmospheres under normal operating conditions. An alternative to these metal-based systems is silicon carbide (SiC) and some perovskite ceramics, such as (La_0.80_Sr_0.20_)_0.95_FeO_3_/Gd_0.1_Ce_0.9_O_2_.^76^Figure 1 demonstrates that SiC can have high electrical resistivity and relatively high thermal conductivity. However, it is important to note that these values are highly dependent on processing conditions, crystal structure, crystallinity, and impurities, which can lead to deviations of multiple orders of magnitude in these parameters. The specific data point used in Figure 1 is a commercial SiC material with a mixture of crystalline phases and a porosity of ∼33%.^71^ Generally, ceramics can be chemically stable and thermally resilient, which could enable them to become a promising material solution for future production of Joule heating technologies. Carbon materials have also been extensively used for Joule-heated synthesis due to their versatility and range of properties. These materials can be produced from various precursors through multiple techniques,^66,77−79^ resulting in significant variations in their electrical and thermal characteristics due to the difference in the formation of carbon structures at both atomic and nanoscale levels; graphite, graphene, carbon nanotubes, activated carbon, carbon fibers, and amorphous carbon each offer unique benefits and exhibit varied material properties. For instance, graphite and carbon nanotubes can provide high thermal conductivity, while graphene offers exceptional electrical conductivity. The selection of carbon type and its specific properties can be optimized for targeted Joule heating applications, enabling a wide array of uses in synthetic processes. Amorphous carbon materials are generally characterized as resistive conductors with reduced thermal conductivities compared to most metals. The increased electrical resistivity enables more efficient conversion of electrical energy into heat, potentially requiring lower current inputs than many other systems. A major drawback of amorphous carbon materials is their susceptibility to oxidative degradation at temperatures as low as 400 to 600 °C. This limitation necessitates the use of inert atmospheres to prevent potential oxidation/degradation of amorphous carbons, which may restrict their application to specific reactions or require specialized reactor designs. Consequently, while carbon materials offer many advantages, to date their use for performing Joule heating reactions at scale is still confined to conditions where these preventative measures can be effectively implemented.

### Reactor Design

The rational design of the Joule heating material for chemical synthesis and advanced material production is crucial for reaching optimal process efficiency. It is important to note that, particularly in the case of chemical syntheses, an active catalytic layer is often integrated within the Joule heating material to enhance the reaction efficiency and selectivity, as the catalyst directly influences the chemical transformation occurring within the heated material. While there are multitudes of methods for incorporating catalytic coats to these materials, these processes are not the focus of this section and therefore relevant discussions are omitted; the reader is directed to a recent review that covers these concepts in great detail if they are interested in these topics.^72^ This section will specifically focus on the design of Joule heating materials to increase process efficiency, including various form factors to optimize accessible surface areas, flow fields, and pressure drop. By tailoring these design parameters, the performance and efficiency of Joule heated processes can be further optimized.

**Figure 2:**
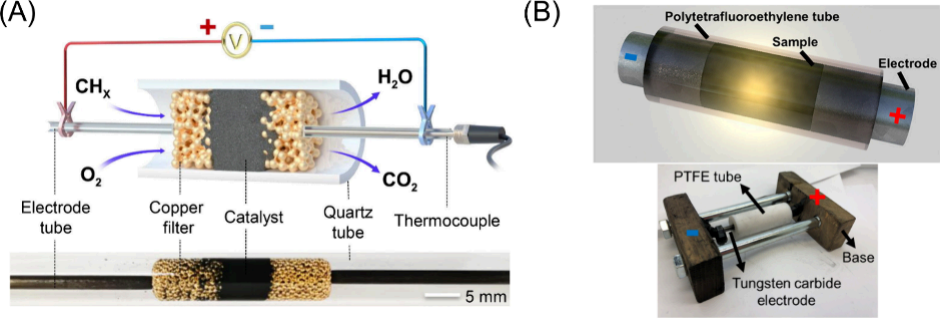
(A) Schematic illustration of the reactor designed by Zhang et al. alongside a photograph of the custom-built reactor. Reproduced with permission from ref (83). Copyright 2023 Elsevier. (B) Generalized illustration, in addition to a digital image, of the flash Joule heating reactor developed for the production of graphene oxide from various precursors. Reproduced with permission from ref (98). Copyright 2021 American Chemical Society.

Gas-phase reactions typically involve flowing gas over catalytically active species at elevated temperatures to convert the gaseous reactants into the desired products. In conventional industrial processes, heat is typically applied to a packed bed of catalyst particles through the combustion of fossil fuels with indirect heat transfer from outside the reactor to its interior.^80−82^ There have been some examples in the literature focusing on the packed bed design concept of Joule heated chemical reaction systems. For instance, Zhang et al. recently demonstrated the design and fabrication of a Joule heated reactor for the catalytic combustion of hydrocarbons which relied on antimony-doped tin oxide (ATO) as illustrated in Figure 2(A).^83^ The reactor was fabricated from a bed of ATO powders placed between conductive copper filters, which were attached to stainless steel electrodes. Direct current was applied to the stainless-steel electrodes which passed through the conductive catalyst bed, generating significant amounts of Joule heat to achieve temperatures of ∼470 °C with a power input of 12 W. This concept has been employed across various catalyst systems for different reactions,^84,85^ while simulation results demonstrated the feasibility of employing mixtures of conductive particles and nonconductive catalysts for Joule heated reactions, including SMR.^86−88^ Although the concept of leveraging a packed bed of conductive catalyst particles to directly produce heat in addition to carrying out the reaction involves a relatively simple and straightforward reactor design, multiple challenges could arise from using packed bed systems. Primarily, the packed bed of particles can result in large pressure drops, reducing the efficiency of the catalytic reaction. A packed bed connected within a circuit for Joule heating is also commonly employed in the flash Joule heating (FJH) process, as illustrated in Figure 2(B), which has been used to produce a wide variety of advanced materials,^89^ including graphene,^90−93^ critical minerals,^94−96^ and metal carbides,^73,97^ from many different precursors; this technique will be described in more detail in Section 4. Typically, FJH processes involve placing a bed of precursor material between two electrodes and applying large voltages to rapidly elevate the temperature by thousands of degrees in fractions of a second, converting the precursor materials into new materials. This versatile process has been demonstrated to be applicable for the large-scale production of functional materials.

**Figure 3:**
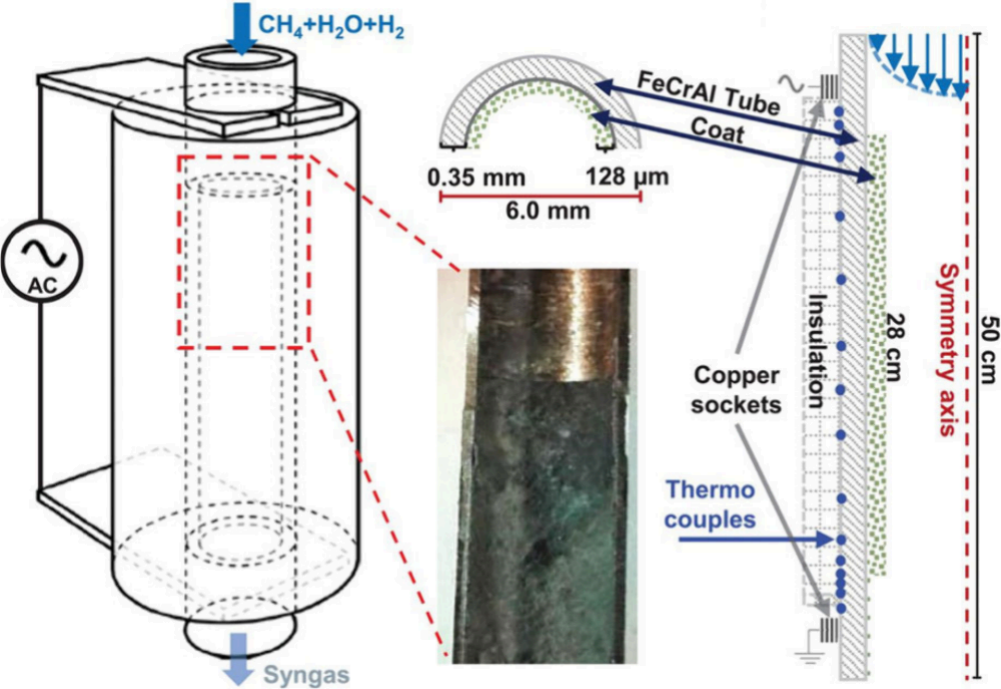
Illustration and optical image of the reactor design employed by Mortensen et al. for the production of hydrogen. Reproduced with permission from ref (59). Copyright 2019 American Association for the Advancement of Science.

Another simple method for developing Joule heated processes, aside from the use of a conductive packed bed, involves fabricating reactors with one or more heating wires, which can be coated with catalytically active species and connected to an electrical power source. In these systems, thermal energy produced via Joule heating as current passes through the wires directly heat the catalytic coating on the wires, enabling efficient conversion of reactants into products. This process has been thoroughly investigated for SMR and many other reactions, including understanding a number of key process parameters, such as input power, reactant concentrations, and catalyst loading content.^99^ Generally, the application of Joule heating, with optimized process inputs, can enable the large-scale production of desired products using reactors that are only a fraction of the size of traditional externally heated systems. In a seminal work, Mleczko et al. demonstrated the successful Joule heating-enabled dry methane reforming using FeCrAl heating elements coated with a LaNi_0.95_Ru_0.05_O_3_ catalyst.^100^ The FeCrAl element was placed in a heat resistant nickel alloy tube, which was shielded by an additional alumina tube to minimize interactions between the process stream and the catalytically active alloy parts for making the reactor. The FeCrAl alloy could be formed into geometries that increase surface area exposed to the reactant stream, thus increasing the efficiency of the process. The wire was capable of achieving temperatures from 700 °C – 900 °C, allowing full conversion with 4 stages of the coated FeCrAl elements. Similarly, Lively et al. have demonstrated that Joule heating can be employed in direct air capture of CO_2_, enabling the energy-efficient release of CO_2_ molecules through desorption.^101^ Specifically, exposing the carbon fiber to 7 V can generate sufficient heat to reach necessary temperatures (∼110 °C) within a minute, enabling more rapid gas desorption compared to externally driven thermal desorption. This work demonstrated great potential for the improved performance of direct air capture CO_2_ systems, which exhibited only a 7% loss of the initial Joule heating input to convection. We note that these processes are not limited to single-fiber structures within reactors but have also been extended to many wires assembled in parallel or screens for the same purposes.^101−103^ Similar to wire-based systems are electrically resistive metal tubes which are coated with catalytic species through different techniques. Using this approach reduces the complexity of the reactors themselves, requiring only the tubing to be directly Joule heated. This advantage has been leveraged in a study by Mortensen et al., who used a FeCrAl tube coated with a zirconia-derived porous washcoat, embedded with nickel species to facilitate the SMR reaction (Figure 3).^59^ Copper sockets were mounted to both ends of the tube which enabled the connection to an alternating current for Joule heating. The tubing had many thermocouples welded throughout the reactor to monitor the temperature at various points, which became higher with increasing distance along the reactor tube, reaching a maximam temperature of ∼800 °C. The simplicity of these systems allows easy design and fabrication, while still exhibiting high efficiencies for electrified methane reforming.^59^ However, wire and tube-based systems lack the ability to greatly alter the accessible surface area of catalytic supporting materials for reactions and to optimize the flow fields within the reactor to improve reaction efficiency.

Alternatively, Joule-heatable monoliths, such as metallic, ceramic, or carbon foams, offer a promising solution due to their relatively high surface areas. These monoliths can be catalytically active themselves or embedded with catalytically active species, providing a more flexible option for various reaction systems. Foam-based systems provide opportunities to incorporate higher levels of catalyst loadings due to their larger surface areas compared to wire/tube-derived reactors.^72^ These foams can also increase mass and heat transfer rates due to the tortuosity of the flow paths created by the open-cell structures. For example, Yu et al. fabricated mesoporous 3D carbon monoliths from polymeric precursors which were subsequently pyrolyzed under inert conditions.^104^ The carbon structure was embedded with silver and cobalt species which served as catalytic species for the oxidation of formaldehyde at elevated temperatures. The reactor was fabricated by inserting the carbon into a quartz tube and clamping the carbon on both sides with copper foams, which were connected to a direct current (DC) power source. Heating the carbon to 125 °C through Joule heating was 7 times more rapid than conventional external heating. The carbon material demonstrated robustness and durability, withstanding up to 100 cycles, illustrating the feasibility of using these materials for Joule-heated catalytic conversions in practical applications when oxidation is not a concern (at low reaction temperatures). Tronconi et al. developed similar reactors derived from SiC foams washcoated with rhodium/alumina catalysts.^105^ The SiC foam was placed in a quartz tube and connected to a power supply by two copper felts which were attached to steel plates. The power required to heat the reactor to temperatures above 700 °C was dependent on the mass of reactants flowing through the system. Power inputs of 386.4 and 489.3 W were required for gas hour space velocities (GHSV) of 100,000 cm^3^/h/g_cat_ and 150,000 cm^3^/h/g_cat_, respectively, and full conversion was achieved under both conditions.

While open-cell foams provide increased mass and heat transfer rates, the continuously developing field of additive manufacturing provides opportunities to even further extend this concept.^106^ Additive manufacturing was originally developed for producing materials into complex geometries for customized applications through a layer-by-layer addition process. Over the past decade, technological advancement has enabled the extended use of additive manufacturing for the production of metals,^107^ ceramics,^108^ and carbon with intricate shapes.^78^ In the context of Joule heating processes, additive manufacturing provides the opportunity to fabriate heaters with controlled and optimized flow paths and/or heat/mass transfer mechanisms. While examples of employing additive manufacturing specifically for the development of Joule heating reactors are still limited, recent works have demonstrated the development of relevant material fabrication processes.^109−112^ Additive manufacturing of metals has been established typically relies on laser-based sintering processes to develop complex geometries. However, the laser sintering process can involve high energy consumption, and the available selection of metal feedstock can be limited. We note a recent approach demonstrated the fabrication of complex metal structures through infusing prefabricated, 3D-printed polymer structures with water-soluble metal precursors. The composite could then be exposed to elevated temperatures to decompose the polymer template while a continuous metal structure retained.^113^ This process was applied to nickel, cobalt, and other metal species, demonstrating the potential of this approach for broadly producing materials suitable for Joule heating applications. Similarly, the production of additively manufactured carbon materials with Joule heating capabilities has been demonstrated through multiple different approaches. These typically include the extrusion of viscous inks which are loaded with large fractions of carbon particles, like graphene oxide,^109,110^ or through the conversion of an additively manufactured polymer precursor to carbon through chemical treatments and subsequent pyrolysis.^79^ This area has recently been further expanded by the successful use of commodity polypropylene precursors through fused filament fabrication to develop carbon with complex geometries.^111,112^ The polymer precursors could be converted to carbon structures after solid-state chemical treatment enabling the production of macroscopic carbon materials that demonstrated efficient Joule heating at low power inputs. These material fabrication methods demonstrate excellent potential for the production of tailorable reactors for future Joule heated syntheses and material production. It is worth noting, for all of the mentioned reactor designs, that additional considerations may be required when scaling to industrial production volumes. Although the process intensification enabled by electrifed heating processes greatly reduces required reactor volumes, various other factors in material and reactor design must be taken into account. For instance, at larger scales thermal expansions and contractions may become much more significant and result in disruption of electrical pathways. This could be circumvented through careful material selection or potentially through careful design of multiple smaller elements connected through the same electrical pathway.

### Chemical Synthesis through Joule Heating

The use of Joule heating has been demonstrated in several established industrial chemical syntheses due to the wide range of performance properties and tunability in reactor design that Joule-heatable materials confer.^36,114^ Through this highly efficient heating strategy, energy-intensive endothermic reactions could be decarbonized. This section discusses several key industrially relevant reactions that significantly contribute to current CO_2_ emissions, including ammonia synthesis and decomposition, CO_2_ reduction, hydrocarbon cracking, and other important processes. A summary of important reactor parameters (joule heating material, active catalyst, reaction performed, reactor design, and operating conditions) for key works is provided in Table 1.

**Table 1:** Summary of Important Design Parameters for Various Joule-Heated Chemical Syntheses

ref	Joule heating material	active catalyst	reaction performed	reactor design	operating conditions
(135)	porous carbon paper	Fe and Ru catalysts	CH_4_ conversion and NH_3_ synthesis	quartz tube	≤2126 °C
					0.02 s intervals
(153)	NiCrAl foam	Ru and Ru/Al_2_O_3_	NH_3_ decomposition	quartz enclosure	450–580 °C
					1000–11000 mL/g_Foam_/h
					128 W/cm^3^
(59)	FeCrAl alloy tube	Ni coated washcoat	CH_4_ conversion	FeCrAl alloy tube	≤800 °C
					50 mbar
(173)	silicon carbide (SiC)	5 wt % Ni deposited on ceramic supports	CH_4_ conversion	quartz tube	≥800 °C
					WHSV of 70–940 h^–1^
(105)	silicon-infiltrated silicon carbide (Si-SiC) foams	Rh/Al_2_O_3_	CH_4_ conversion	stainless-steel tube, Cu foil, ceramic tube, quartz tape	≤750 °C
					GHSV of 100 000 and 150 000 cm^3^/h/g_cat_
(181)	SiSiC foam	Rh/Al_2_O_3_	CO_2_ conversion	stainless-steel tube, ceramic tube	≤800 °C
					GHSV of 100 to 600 kNl/kg_cat_/h
(182)	FeCrAl support	Ni-type catalyst	CO_2_ conversion	FeCrAl support	1050 °C
					10 barg
(186)	porous carbon felt		depolymerization	quartz tube, bilayer of porous carbon felt, Cu foil	≤600–1050 °C
					transient heating duration ∼0.11s
					26 V
(190, 191)	graphite-like carbon	Fechral catalyst	conversion of ethane and ethylene	T-shaped reactor	≤1000 °C

#### Ammonia Synthesis/Decomposition

Ammonia (NH_3_), with 175 million metric tons (Mt) produced in 2020,^115^ has long been a prominent industrial chemical due to its significant usage to produce fertilizers (∼80% of all NH_3_ is used in agriculture) as well as cleaning solutions, refrigerants, nitrogenous compounds, and fuels.^116^ With a global market of $69 billion in 2021, NH_3_ production is projected to reach $224 billion by 2050 in part due to its anticipated role as a hydrogen carrier.^117^ While hydrogen gas represents a promising carbon-free energy resource, issues with hydrogen storage and transport encourage the use of carrier molecules.^118−120^ Due to ammonia’s promising role as a hydrogen carrier, the synthesis and decomposition of NH_3_ are critical reactions that require sustainable manufacturing processes, which can significantly benefit from electrification through Joule heating, enhancing efficiency and reducing environmental impact. First developed through the Haber-Bosch process, which has since undergone extensive optimization, the conventional synthetic approach for ammonia requires high pressures (∼300 bar) and elevated temperatures (∼500 °C) in the presence of a catalyst.^114,121,122^ The use of iron-based (Fe),^123−128^ and more recently ruthenium-based (Ru) catalysts,^129−132^ deposited on support materials has been extensively investigated to improve reaction efficiencies. However, several challenges still exist including relatively high costs, poor catalytic stabilities, and sluggish reaction rates.^132−134^ The high reaction temperatures necessary for the synthesis of NH_3_ also led to increased catalyst-particle sintering effects.^132^ Moreover, metal hydrides, which play an important role in NH_3_ production, have been shown to hinder catalyst stability.^133,134^ It has been found that the implementation of Joule-heating to NH_3_ synthesis provides several advantages, particularly associated with the ability to rapidly heat and quench reactions with programmable electric currents. For example, Hu et al. studied the use of a carbon felt heater to develop a nonequilibrium, thermochemical synthesis technique with a wide range of accessible temperatures (up to 2,126 °C) and rapid pulsed heating (0.02 s intervals).^135^ This strategy resulted in excellent reaction selectivity and catalyst stability for the pyrolysis of methane as well as NH_3_ synthesis. Specifically using a Fe catalyst, an optimized synthesis rate of 6000 μmol g_Fe_^–1^ h^–1^ was achieved for time-on-streams greater than 100 h at ambient pressure. The use of a Ru-based catalyst was also demonstrated in this system, achieving similarly high reaction rates of 4000 μmol g_Ru_^–1^ h^–1^ for over 100 h. As the time scale for migration of Ru catalysts to the carbon felt required 4–8 s, the rapid heating (∼0.11 s) up to 1,400 °C enabled by Joule heating prevented the sintering of the catalyst and led to high catalyst stability. The excellent selectivity and reaction rate imparted by the rapid heating and quenching, as well as the programmable nature of Joule heating, demonstrate its ability to not only decarbonize chemical syntheses but also improve fundamental mechanistic parameters.

**Figure 4:**
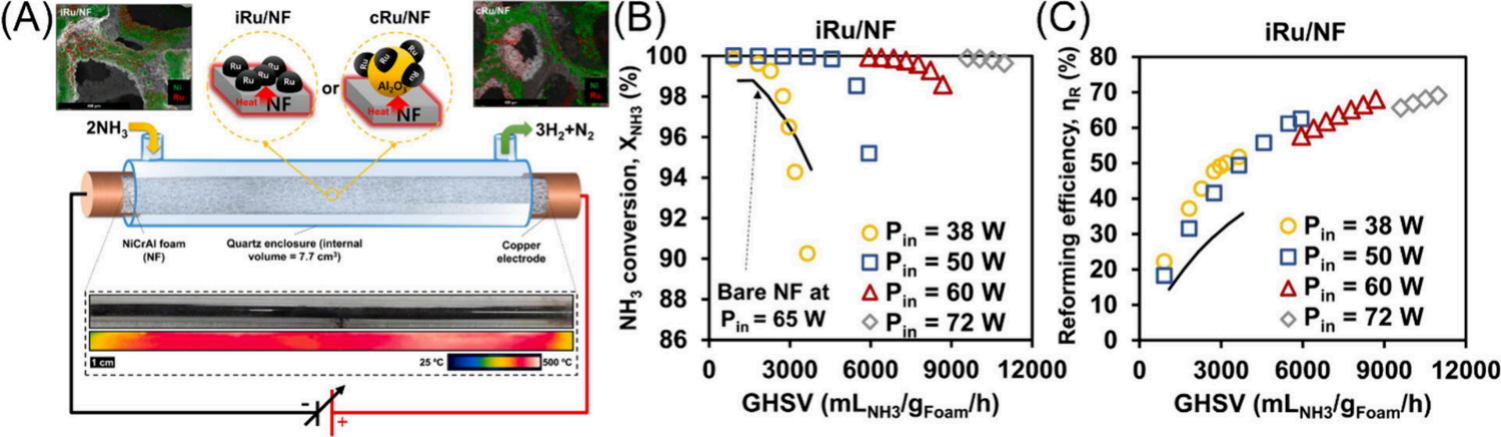
(A) Schematic demonstrating NH_3_ decomposition with a NiCrAl foam catalyst support coated with Ru/Al_2_O_3_. (B) NH_3_ conversion and (C) reforming efficiency as a function of gas hourly special velocity at various input temperatures. Reproduced with permission from ref (153). Copyright 2021 Elsevier.

As previously discussed, the application of NH_3_ for hydrogen storage and transport is highly promising,^136^ as the existing NH_3_ infrastructure is well-established due to its longstanding utility. Furthermore, NH_3_ can be liquified at relatively low pressures and temperatures in addition to having a high hydrogen content (17.8 wt %).^137−139^ The portable use of NH_3_ to prepare H_2_ is only feasible if several key challenges can be addressed.^136,140^ First, the decomposition of NH_3_ is a highly endothermic process which necessitates high reaction temperatures. With an optimized catalyst system, a temperature of 500 °C was necessary to achieve a high conversion rate (>99.9%).^141^ When considering the application of H_2_, high purity is an important requirement as low concentrations of ammonia (0.1 ppm) have been shown to irreparably degrade processes such as proton-exchange membrane fuel cells.^142^ Conventional ammonia crackers utilize nickel-based catalysts supported on aluminum oxides with external energy sources that reach ∼900 °C to primarily anneal and galvanize metals.^140^ Though there have been several methods of efficient heating for NH_3_ decomposition, most rely on process intensification through microreactor designs and coupling of oxidation-decomposition reactors.^143−148^ Recently, the use of plasma has been investigated with several strategies displaying the successful production of H_2_, though these could result in low energy efficiencies and high power consumption.^149−152^ The use of Joule heating for NH_3_ decomposition has also been demonstrated by Jo et al. where a nickel–chromium aluminum (NiCrAl) foam was used as a Joule-heatable catalyst support with a Ru/aluminum oxide (Al_2_O_3_) coat.^153^Figure 4(A) shows a small-scale prototype reactor developed with an internal volume of 7.7 cm^3^ in order to enhance powder density and reaction efficiency while minimizing heat-transfer limitations. Specifically, a powder density of 128 W/cm^3^ was demonstrated with an operating temperature between 450 and 580 °C, leading to a hydrogen production rate of 1.56 cm^3^ H_2_/s/cm^3^ reactor with a high reforming efficiency of 69.2%. The conversion and reforming efficiency are shown as a function of flow rate for several input powers in Figures 4(B) and 4(C), respectively. The bare foam with the absence of a catalyst was found to have high conversion (∼95%) at high input powers while exhibiting lower catalytic activity. For the Ru/Al_2_O_3_-coated NiCrAl foams, an increase in power input generally resulted in increased conversions at constant flow rates. Moreover, the specific activity of the catalyst was found to be increased by a factor of 10 through Joule heating compared to an external heating method. Through electrification of the heating process, the synthesis and decomposition of NH_3_ can be efficiently achieved to not only drive decarbonization efforts, but also expand the utilization and distribution of this critical chemical.

### Steam Methane Reforming (SMR)

As discussed in the previous subsection, hydrogen is a central element of global decarbonization due to its potential as a carbon-free energy source.^154−156^ In addition, H_2_ serves several economic sectors including oil refining, ammonia and methanol synthesis, steel production, and hydrogen cracking processes.^157,158^ Though it has a vital role in developing a sustainable future, the widescale implementation of H_2_ may be obstructed by current production processes.^159^ For example, nearly all H_2_ production (95%) uses fossil fuels.^160^ The most prominent H_2_ production strategy is SMR, which produces 48% of the global hydrogen supply.^161,162^ Specifically, SMR involves two processes: the endothermic steam methane reforming reaction and the exothermic water–gas shift reaction.
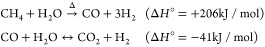


In the first step, oxidation of methane’s carbon atom results in stripping of hydrogen which is then followed by the further oxidation of the carbon atom to produce an additional H_2_ molecule. The resulting gas mixture of primarily H_2_ and carbon monoxide is often referred to as syngas. As this overall process is highly endothermic, this reaction takes place at 700–1000 °C under 0.3–2.5 MPa pressure.^38,59,163^ A significant amount of heat is supplied by the combustion of methane feedstock and off-gases, resulting in the production of 8.6–9.3 Mt of CO_2_ per Mt of H_2_ produced through conventional SMR processes, of which, combustion of hydrocarbons to heat this reaction accounts for approximately 17–41% of emissions.^164−166^ In industrial sectors, this process involves the combination of serveral hundreds of 10–14 m long tube reactors heated by gas burners which are periodically positioned to distribute heating.^38,167^ However, due to poor thermal conductivities of reactor walls and catalysts, gas burners must reach significantly higher temperatures to achieve the required heat flux inward to reaction sites. Generally, ∼ 50% of heat is transferred to the reaction and the rest leaves as hot flue gas as latent heat.^168^ Catalysts take up ∼2% of reactor volumes as intrinsic catalytic activity, while the ability to heat across catalysts is an important consideration as temperature gradients can result in carbon formation and poor utilization of catalyst.^163,169^ Additional challenges with conventionally fired reactors include long startup/shutdown times, heat transfer nonuniformity/inefficiencies, and several side reactions due to nonselective heating including coke formation and catalyst-particle sintering.

The electrification of SMR through Joule heating represents a disruptive technology to decarbonize H_2_ production by not only incorporating more efficient heating that can directly heat reaction sites, but also having the capability of integrating renewable energy sources.^170^ The use of Joule heating for SMR has been demonstrated by Spagnolo et al. in 1992 using a 316 stainless steel screen washcoated with a Ni-Alumina catalyst, though the metal screen experienced significant heat-related embrittlement.^103^ Kameyama et al. studied the use of Al/FeCrAl/Al clad plate Joule heating elements impregnated with Ni-based catalyst for SMR, leading to the methane conversions as high as 97%.^57,171^ However, this method faced challenges due to the spalling of the active surface, which reduced to just 36% of the original surface area after 50 h at 700 °C. Mortensen et al. investigated the electrification of SMR using a FeCrAl-alloy tubular reactor with a Ni-based catalyst washcoat.^59^ As described in the previous section, copper sockets were placed at opposite ends of the tube, attached to an alternate current power source, and temperature profiles were measured during reaction from various thermocouples welded across the tube reactor. They found temperatures increase rapidly prior to approaching the inlet and the catalyst coated zone, which then slightly drop at the coated zone due to the onset of the endothermic SMR reaction. Following this drop, the temperature profile is nearly linear across the coated zone indicating a significant amount of heat was being utilized for the SMR reaction. With an outlet temperature of 800 °C at 50 bar, conversion of methane reached 87% with thermodynamic equilibrium being approached rapidly. This nearly constant heat flux across the catalyst resulted in an average catalyst utilization of 20%, which is an order of magnitude higher than the conventional SMR reactor heated by a gas burner furnace. While this work used only a single tube, extrapolation to several SMR tube reactors was found to significantly reduce required reactor volumes. For example, a ∼ 5 m^3^ electrified reformer can replace a conventionally fired reformer that produced 2,230 kmol H_2_/h at a conversion of 75.4% which required a total volume of 1100 m^3^. In a subsequent study,^58,172^ the performance of these electrified tube reactors was found to be primarily governed by external diffusion. To promote this gas–solid mass transfer, the use of small diameter tube reactors is preferred, including microchannels or honeycomb monoliths. Palma et al. demonstrated SMR with the use of silicon carbide-based heating elements dip coated with Ni-based catalysts.^173^ This reactor design was capable of reaching over 700 °C, though conversions were limited to the kinetic regime. Additionally, a specific energy demand of 5 kWh/Nm^3^_H2_ was reported which indicates hindered thermal efficiency.^173^ Furthermore, Tronconi et al. demonstrated the electrification of SMR with silicon-infiltrated silicon carbide (Si-SiC) foams coated with rhodium (Rh)/Al_2_O_3_.^105^ Direct Joule heating of SMR was demonstrated up to 750 °C with nearly full methane conversion that approached equilibrium for a series of reaction conditions. High energy efficiencies (up to 61%) and low specific energy consumptions (2 kWh/Nm^3^_H2_) were found at 650 °C with a 150,000 cm^3^/h/g_cat_ gas hourly space velocity (GHSV), attributed to the interconnected geometry and bulk resistivity of the Si-SiC foam. The electrification of SMR through Joule heating coupled with the use of renewable energy sources represents a key chemical process that can drive decarbonization efforts in the chemical industry.

### CO_2_ Conversion/Reduction

Closely intertwined with reducing CO_2_ emissions, the reforming of CO_2_ to synthetic gas represents an important strategy to valorize CO_2_ and further reduce its associated environmental impacts.^174,175^ CO_2_ reduction through the reverse water–gas shift (RWGS) and dry methane reforming are shown below:
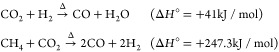


While both are promising routes to prepare mixtures of CO and H_2_, these reactions also represent highly endothermic reactions that require high operating temperatures.^176,177^ Similarly to other industrial scale chemical syntheses, the electrification of CO_2_ reforming is a highly promising decarbonization strategy as it can take advantage of CO_2_ feeds while producing value-added syngas.^178^ Though this could serve as a multifaceted decarbonization pathway, the commercialization of these technologies is still in progress.^179,180^ One of the most mature electrified CO_2_ valorization approaches involves the use of a solid oxide electrolyzer for electrochemical reduction, however, a large specific energy demand is required (6–8 kWh/Nm^3^_CO2_).^180,181^ Mortensen et al. studied the electrification of the RWGS reaction using a Ni catalyst on a FeCrAl support.^182^ Through Joule heating, temperatures of 900–1050 °C were achieved to convert a feed of H_2_/CO_2_ ratio of 2.25 at 10 bar to syngas with the following composition: 46% H_2_, 23% CO and 8% CO_2_. Notably, the Joule heated reaction demonstrated higher catalytic activity, nondetectable methane slip, and suppression of carbon formation at these high temperature conditions.

**Figure 5:**
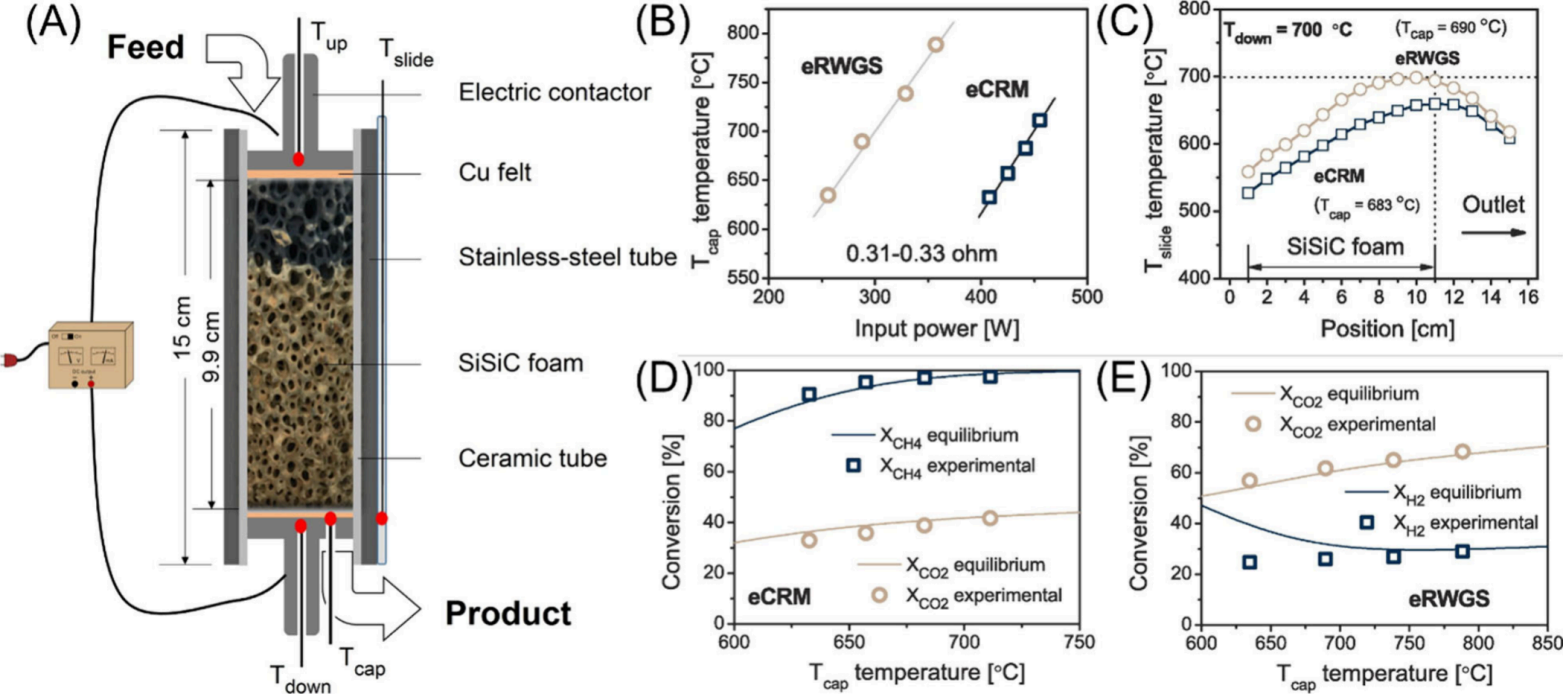
Joule heating-enabled RWGS and CRM. (A) Schematic illustration displaying the reactor apparatus using open cell SiSiC foams. (B) Measured temperatures for RWGS and CRM as a function of input power. (C) Temperature profiles for the reactor axial wall and outlet for both RWGS and CRM. Conversion of CO_2_ and CH_4_ as a function of reactor temperature for (D) CRM and (E) RWGS. Reproduced with permission from ref (181). Copyright 2023 Elsevier Chemical Engineering Journal.

In another work, Tronconi et al. investigated the use of open cell silicon-infiltrated silicon carbide foams coated with Rh/Al_2_O_3_ for both RWGS and CO_2_ reforming of methane (CRM) as illustrated in Figure 5(A).^181^Figure 5(B) shows the temperature as a function of input power during both RWGS and CRM. The porous scaffolds were found to exhibit excellent Joule heating properties that resulted in a wide operating temperature range of up to 700 °C, due to both the resistivity of the foam and its interconnected geometry. Moreover, a near linear relationship between the measured temperature and input power was observed following the Joule law. Specifically, the temperature profiles of the axial wall and outlet are shown in Figure 5(C) for both RWGS and CRM where temperature was observed to increase along with foam reactor length. A maximum temperature was reached at the bottom of the foam reactor for both RWGS and CRM, which then immediately fell as the outlet was approached. This same trend was observed for all investigated reaction temperatures. The CO_2_ and CH_4_ conversion are shown in Figures 5(D) and 5(E) as a function of temperature for CRM and RWGS, respectively. It was observed that this Joule heating-driven process could achieve high CO_2_ conversion which approached thermodynamic equilibrium for both reactions. Moreover, this process can achieve a high CO_2_ conversion with low specific energy demands, calculated to be 0.7 kWh/Nm^3^_CO2_ for an optimized process configuration (assuming a 90% recovery of sensible heat and 95% overall adiabacity). Additionally, the use of water electrolysis-derived H_2_ feedstocks was also calculated to achieve significantly lowered specific energy consumptions (4.5 kWh/Nm^3^_CO2_) compared to solid oxide electrolyzer strategies. When reaction temperatures were above 675 °C, no detectable CH_4_ was observed. Overall, a conversion of 94.2% with a H_2_ rich feed and excellent electrical and catalytic stability up to 75 h was achieved with the Joule heated apparatus. Ni-based catalysts have also been used for electrified dry methane reforming (DMR). Palma et al. studied two different types of carriers: a silicon-SiC open-cell foam and a SiC honeycomb monolith, employing both microwave and Joule heating methods.^183^ It was found the honeycomb monolith exhibited improved microwave heated catalysis as the foam’s voids resulted in reduced heat adsorption and increased specific energy consumption. However, through Joule heating, the foam had a lower energy consumption rate (2.6 kWh/Nm^3^_H2_), which approached theoretical values (1.90 kWh/Nm^3^_H2_) and was approximately one-quarter of the energy consumption of microwave heating reactions.

A main limitation of DMR, compared to SMR, is the expensive catalyst requirements and comparatively low hydrogen yields, though the CO-rich syngas products demonstrate its promising nature within the decarbonization landscape.^184^ Mleczko et al. used a Fe–Cr–Al alloy heating element coated in LaNi_0.95_Ru_0.05_O_3_ catalyst, resulting in fairly moderate (29.4%) methane conversion at 900 °C, possibly due to low catalytic surface area.^100^ Vlachos et al. used a commercial carbon fiber paper coated with a PtNi/SiO_2_ catalyst for DMR and compared conventional heating to rapid pulse Joule heating with heating rates up to 14000 °C/s.^185^ They found that their millisecond-pulse heating profile doubled the energy efficiency compared to conventional heating as well as increased catalyst stability and resulted in a higher H_2_/CO ratio. The dynamic electrified reaction led to an increased methane conversion (67%) compared to conventional heating (40%). It was also found that the rapid pulse heating created an *in situ* catalyst regeneration process, where catalyst stability was improved due to the suppression of detrimental phase segregation, coke formation, and catalyst self-sintering.

### Other

There are several emerging uses of Joule heating in various chemical synthesis and production processes. For example, Hu et al. demonstrated the use of Joule heating for the depolymerization of commodity plastics.^186^ A bilayer of porous carbon felts was utilized to develop a thermochemical, nonequilibrium depolymerization technique by coupling a temporal heating profile with a spatial temperature gradient. The top layer was first Joule heated, which then transferred heat down to the underlying carbon felt and a plastic reservoir. This temperature gradient promoted a series of behaviors, including plastic melting, wicking, and reaction. As the plastic encountered higher temperatures as it traversed the carbon felt bilayer, a significant amount of thermal triggered depolymerization was achieved. This behavior was paired with a pulsed Joule heating to minimize undesirable side products. The upcycling commodity polyethylene terephthalate (PET) and polypropylene (PP) could be directly converted to monomers without the use of a catalyst, reaching yields of 43% and 36%, respectively. Compared to a catalyst-free depolymerization approach via conventional heating at near equilibrium conditions that exhibited a yield of ∼10%, this catalyst-free approach demonstrated a significantly higher propene monomer yield of 36%.^186^

Light alkenes represent a key chemical building block as a wide variety of industrial products use them extensively, ranging from chemical intermediates, polymers, and oxygenates.^187,188^ Ethylene, for example, is mostly produced through the endothermic steam cracking of ethane, naphtha, and petroleum at temperatures above 850 °C, though due to the large carbon-intensive energy input and complex product distillation requirements, other synthetic routes are desired.^189^ Shlyapin et al. recently used Fe–Cr–Al alloy as both a joule heater and catalyst to achieve a high oxidative coconversion of methane and ethane feed gas mixtures to ethylene at 1000 °C through methane dehydrogenation and radical coupling.^190,191^ They found graphite-like carbon deposits covered the surface of the catalyst, which resulted in additional catalytic effects to produce C_3_ and C_4_ hydrocarbons. Still, methane-to-ethylene conversion using Joule heaters, especially impregnated/coated with protective coatings and catalysts, remains largely underexplored.^192^ An alternative approach to energy-efficient light alkene synthesis involves the nonoxidative dehydrogenation of light hydrocarbons, though this endothermic reaction has limited conversions, high reaction temperatures, and significant CO_2_ emissions.^193−195^ Liu et al. investigated the use of a siliceous zeolite-supported catalyst and a carbon molecular sieve hollow fiber membrane which is H_2_ permeable for electrified alkane dehydrogenation.^196^ Through Joule heating of the carbon membrane, suitable conversions were achieved at reaction temperatures ∼150 °C lower than conventional reactors which suppressed coke formation and allowed for exceptional catalyst stability. The electrified membrane reactor was found to reduce CO_2_ emissions by 20% and increase propane conversion by ∼3 times of the equilibrium conversion.

#### Advanced Material Synthesis

Various Joule heating reactor designs have been employed to synthesize high-value materials including graphene, critical metals, metal oxides, and metal carbides from a wide range of low-cost precursors. While these processes can be carried out through multiple mechanisms, in general employing Joule heating for advanced material synthesis involves short pulses of high voltages and currents to generate temperatures >1000 °C in a fraction of a second. This rapid heating process can enable the direct conversion of precursors of the vaporization and subsequent deposition of new materials which nucleate and grow on the Joule heater surface. This section provides a detailed overview of materials synthesized through these processes and their formation mechanisms in addition to perspective of their potential applications.

### Graphene

The synthesis of graphene and other carbon-based materials through direct Joule heating is a recent advancement that has received significant attention in recent years, primarily focused on FJH.^197−199^ In the FJH method, a carbon-rich sample is pressed between two electrodes and contained within a quartz or ceramic tube under atmospheric conditions or, alternatively, under a slight vacuum condition to facilitate outgassing.^89^ Then, a capacitor bank produces an electric discharge with large voltages which heats the carbon materials to temperatures over 3,000 °C in milliseconds, ultimately converting the raw material to graphite, graphene, other carbon allotropes, or other carbon materials.^89,200^ The FJH synthesis of graphene is advantageous because it does not rely on catalysts, solvents, or inert atmospheres to produce high quality graphene materials, greatly simplifying the production process.^91^

**Figure 6:**
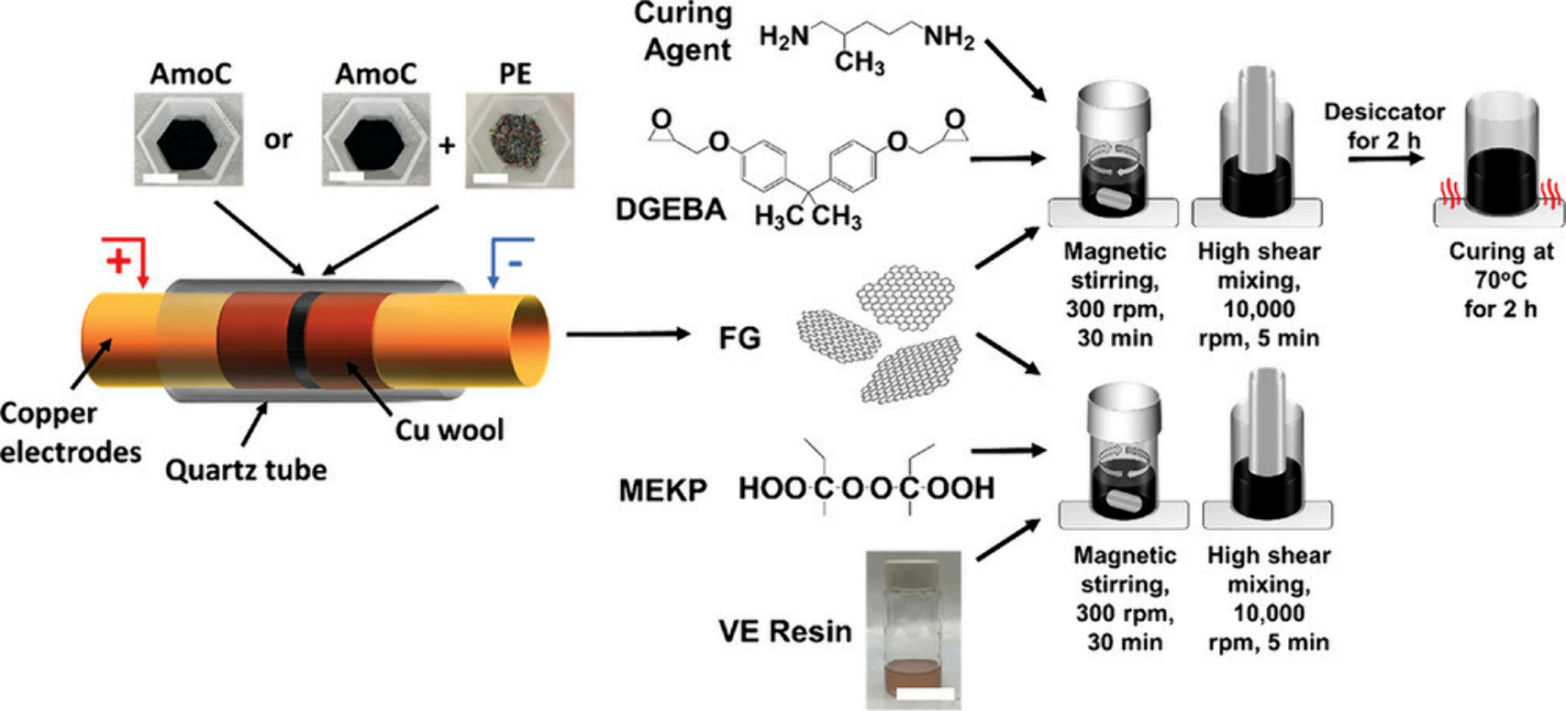
FJH of amorphous carbon and a mixture of amorphous carbon and waste polyethylene to generate FG as an additive to improve the mechanical properties of epoxy and vinyl ester resins. Reproduced with permission from ref (202). Copyright 2023 John Wiley and Sons.

The synthesis of graphene from a variety of feedstocks has been explored, including anthracitic coal, charcoal, calcined coke, pyrolysis ash, waste rubber, waste plastics, carbon black, food waste, CO_2_-derived carbon, etc.^89−92,201,202^ An illustration displaying the conversion of amorphous carbon feedstock to graphene utilizing the FJH process can be found in Figure 6. All of the listed feedstocks were successful in generating turbostratic flash graphene; however, for nonconductive carbon sources, the addition of a conductive material, such as carbon black, is necessary to enable the application of Joule heating.^92,202^ The maximum temperature and reaction time of the FJH process are critical parameters that determine the properties of the synthesized graphene products. In a seminal work in this field, Tour et al. demonstrated that the application of <90 V produced temperatures < ∼ 2700 °C to carbon black precursors, resulting in graphene that contained a large number of defects as evidenced by Raman spectroscopy.^89^ By increasing the voltage applied to the sample, the temperature can exceed 2800 °C and the resulting graphene products exhibit virtually no evidence of defects from Raman spectroscopy experiments. Additionally, longer exposures time (150 ms compared to 10 ms) of FJH process can result in the stacking of multiple layers, decreasing the quality of the graphene product. This could also be mediated by enhancing the cooling rates of the reactor system through using reactor tubes with thinner walls among other techniques. It is also worth mentioning that carbon has a high sublimation temperature of ∼3600 °C which enables the synthesis of highly pure carbon species due to the volatilization of other elements during the FJH process. The morphological evolution of the graphene produced during FJH synthesis was further investigated in a following study.^203^ More specifically, graphene was synthesized from carbon black precursors, and it was found that there were two different populations of graphene morphologies. After the FJH process, it was determined that the resulting graphene existed as sheets of turbostratic graphene or wrinkled graphene through transmission electron microscopy. By increasing the exposure time of the FJH pulse, the relative amount of turbostratic sheets compared to wrinkled graphene increased until reaching ∼200 ms. After which, the graphene composition remained unchanged. Furthermore, it was found that short pulses ensured rotationally decoupled morphologies, but longer durations allowed for the formation of graphite-like morphologies as the individual layers had sufficient time to stack upon one another. Through this work, it was suggested that the optimal duration of the FJH process is ∼30–100 ms to produce high quality turbostratic graphene. However, it has also been demonstrated that the purposeful incorporation of defects into graphene products could be leveraged to enhance their utility in multiple applications. Cao et al. used graphene oxide as a precursor which was reduced through the FJH process to create a graphene structure with inherent defects, which increased their lithium ion storage capacity.^93^ The FJH process removed adsorbed water, in addition to epoxides, hydroxyls, and carbonyls from the graphene oxide precursor which resulted in the evolution of gaseous byproducts and the separation of the graphene oxide layers and disruption of the conductive pathway. Once the gases were removed, the graphene layers came in contact again, and the FJH proceeded, decreasing interlayer distances and the formation of a 3D-reduced graphene oxide network with large numbers of defects. The presence of defects in the graphene resulted in their increased capacity for serving as lithium-ion battery electrodes. These studies confirm that FJH for graphene synthesis is applicable to a wide variety of carbon-based materials, however, it should also be noted that changing the FJH parameters often alters the physical characteristics and quality of the graphene samples significantly. These parameters include the mass of the sample per batch, the time scale of the FJH, the electrical resistance of the precursor, applied voltage, and the reactor tube dimensions.^201,204^ Therefore, further optimization of parameters is required to produce graphene with consistent morphologies, regardless of the feedstock utilized. The graphene materials produced through the FJH process have also demonstrated significant utility in multiple applications, including the aforementioned example as electrodes in batteries, and fillers in polymer nanocomposites. Specifically, Figure 6 depicts the use of amorphous carbon and waste polyethylene as precursors for graphene synthesis.^202^ This work investigated the application of synthesized graphene products for improving the mechanical properties of vinyl ester and epoxy-derived thermoset polymers. The presence of the waste-derived graphene increased the Young’s modulus and hardness of the composites by up to 73% for both properties compared to the neat polymer.

Other carbon materials, such as heteroatom doped graphene or carbon, carbon allotropes (such as nanodiamond and concentric carbon), alloy nanoparticles, carbon nanotubes, etc. can also be synthesized through Joule heating methods.^198,200,205−209^ In a study by Chen et al., carbon black was Joule heated with the addition of a variety of dopants (oxides, organic compounds, and elements) to generate single element, two element, and three element heteroatom-doped graphene.^205^ Boric acid, melamine, poly(1,4-phenylene sulfide) and red phosphorus were investigated as heteroatom dopants for the graphene materials. These dopants were mixed with the carbon precursor at various ratios to maximize the doping contents and subjected to the FJH process. The maximum heteroatom content was 1.8 at%, 5.4 at%, 1.5 at%, 5.5 at%, and 1.6 at% in the boron, nitrogen, sulfur, phosphorus, and oxygen doped materials, respectively. Despite the incorporation of different heteroatoms into the carbon lattice, the doped materials still exhibited high graphene quality, turbostratic structures, and expanded interlayer spacings. The difference in electronegativity of the heteroatoms compared to the carbon atoms in the framework resulted in materials with altered functionality that could be leveraged in electrocatalysis and electrochemical energy storage materials. Additionally, Chen et al. were able to synthesize fluorinated nanodiamonds, fluorinated concentric carbon, and fluorinated graphene through FJH by employing a variety of fluorine-containing reactants (such as polyvinylidene fluoride and polytetrafluoroethylene) and altering the flash duration (35 to 500 ms).^200^ The resulting nanoparticles were densely packed and well dispersed within the porous carbon substrate, with very small particle sizes of approximately 3.2 nm. Carbon nanotubes (CNTs) were synthesized from carbonized silk fabric loaded with metal salts and Joule heated with a pulse voltage of 40 V for approximately 50 ms.^208^ The resulting CNTs were generated radially along the carbonized silk fabric’s surface with uniform density. Joule heating methods require low energy, and minimal reaction times to form intricate carbon structures, while continued optimization of the Joule heating techniques is crucial to obtain consistent carbon product quality and to scale the reactions for industrial applications.

### Metals and Metal Oxides

In addition to carbon-based advanced materials, Joule heating processes can also be employed to produce metallic or metal oxide species. This is generally accomplished by loading a material capable of Joule heating with metal precursors, which are then subjected to large amounts of heat for their conversion to functional metal and/or metal oxide nanoparticles. One of the initial works in this area demonstrated the successful synthesis of palladium, gold, iron, and tin nanoparticles deposited on carbon nanofibers through a rapid Joule heating process.^210^ This was accomplished by loading metal salts onto carbon nanofiber felts, which were then connected to a power source. This setup enabled heating to temperatures as high as ∼2700 °C in fractions of a second, effectively decomposing the metal precursor. Palladium based precursors were employed as a model system to investigate the impact of processing parameters on the deposition of palladium nanoparticles during the heating process. Specifically, the carbon felt was dipped into an aqueous solution of palladium chloride and then dried prior to the heating process. The metal loaded fibers were then heated to temperatures above 1700 °C for varying Joule heating times and rapidly cooled upon the removal of electrical current. Noteworthily, the study found that short, pulsed heating limited the diffusion and migration of palladium nanoparticles as they formed on the carbon nanofiber surface, resulting in uniform nanoparticle distributions. Additionally, the size of the nanoparticles was dependent on the heating duration: short exposure to Joule heating (5 ms) produced particles around 4 nm, while longer exposure time (1 s) resulted in much larger particles, approximately 27 nm in size. Furthermore, this process introduced multiple defects within the crystalline structure of the palladium nanoparticles, which can enhance their electrocatalytic activity; the reported method was extended to multicomponent systems, specifically high entropy alloys consisting of up to 8 different metals, using rapid Joule heating processing methods.^211^

Synthesis of metal alloys from multiple components with distinct physical and chemical properties can be very challenging. However, by coating carbon nanofibers with solutions containing multiple water-soluble metal precursors, it was demonstrated to produce alloy nanoparticles composed of platinum, palladium, nickel, cobalt, iron, gold, copper, and tin metals, which exhibit vastly different melting temperatures, atomic radii, and preferred crystal structures. We note that the rapid heating and quenching process in Joule heating can prevent phase separation and result in uniform alloy nanoparticles. Additionally, as illustrated in a previous work, the rapid Joule heating approach produced metallic particles which were tens of nanometers in size. The increased surface area, attributed to their nanosize effects, can enhance their site availability for catalytic reactions, improving the efficiency and performance of the synthesized materials. Kim et al. found that first functionalizing the carbon nanofibers with titanium oxide species through sol–gel chemistry can greatly mediate the high entropy alloy nanoparticle formation process without sacrificing their performance for the reduction of CO_2_ into CO.^211^ It was found that the incorporation of metal oxide species on the Joule-heated fiber surface binds the metal precursor to the oxide surface during the heating process, preventing them from diffusing across the surface of the fiber and nucleating into larger particles. The resulting nanoparticles were smaller than 3 nm in size with high areal densities, which is considered optimal for increasing the systems catalytic activity. Moreover, Qin et al. demonstrated the *in situ* synthesis of a metal oxide anode through a Joule heating-based technique.^212^ This work employed a metal organic framework precursor (ZIF-67) which was blended into a slurry of conductive carbon black (Super P) and a carboxy methyl cellulose (CMC) binder and subsequently fixed to copper current collectors. The composite materials were exposed to voltages up to 50 V in order to generate sufficient heat to decompose the ZIF-67 into cobalt and cobalt oxide nanoparticles. It is important to note that the ZIF-67 is not electrically conductive and this process requires the presence of Super P additive for Joule heating. The system was heated up to 720 °C (31.5 V) to completely decompose the ZIF-67 precursor, which was ultimately converted to porous anodes containing 10 nm −15 nm cobalt oxide nanoparticles through Joule heating for ∼0.2 s–0.3 s. When employed in lithium-ion batteries, these anode materials exhibited high capacities (reaching 1503.7 mA h/g) and excellent cyclability due to the porous structure developed from the Joule heating process, the small particle size of the metal/metal oxide nanoparticles, and the inclusion of nitrogen heteroatoms into the carbon materials due to the high nitrogen contents of the ZIF-67 precursor.

### Metal Carbides and Other Materials

Metal carbides are metallic materials in which carbon atoms are distributed within the densely packed host lattice. The presence of carbon atoms within metallic species can alter the electronic, mechanical, and chemical properties compared to the host metal material.^213,214^ Generally, metal carbides have tunable chemical identities, as well as tailorable surface functionalities that enable their use for a vast range of applications including energy storage,^215^ electromagnetic interference shielding,^216^ water purification,^217^ and catalysis.^218^ Typically, metal carbide synthesis involves carburization of metals with gaseous carbon precursors or high temperature sintering of metals with graphitic carbon materials.^219^ These methods can result in large particle sizes which reduce the efficacy of the materials for various applications, including as heterogeneous catalysts, and can also cause the deposition of coke on the carbide surfaces which can further reduce their utility.^220^

**Figure 7:**
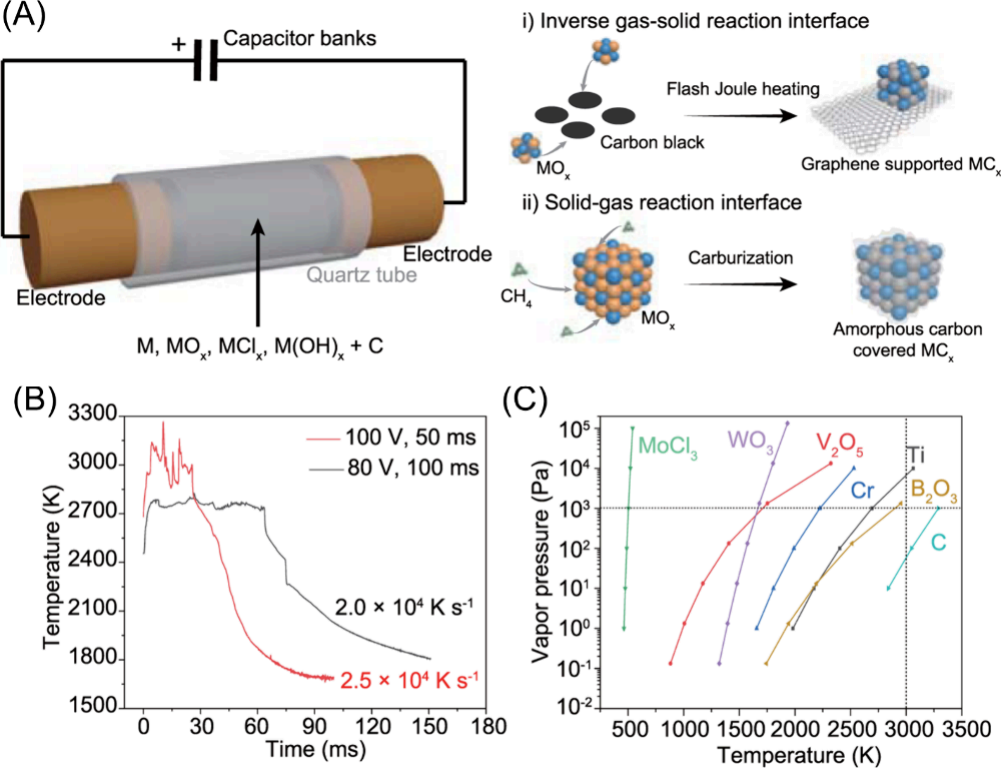
(A) Schematic illustration of the reactor employed for the Joule-heated synthesis of metal carbides in addition to a comparison between (i) the process employed in this work and (ii) traditional synthetic methods. (B) Temperature produced by the Joule-heated reactor as a function of time and applied voltage. (C) Temperature and vapor pressure relationships of the metal precursors and carbon materials. The vertical dashed line represents 3000 K, and the horizontal dashed line represents a vapor pressure of 1 kPa. Reproduced with permission from ref (97). Copyright 2022 Springer Nature.

Joule heating processes have been effectively used to synthesize metal carbide nanocrystals while preventing coke deposition during the synthesis. This method ensures the purity and quality of the nanocrystals by avoiding unwanted carbon buildup. Specifically, Tour et al. have employed FJH to synthesize 13 different carbide materials with controlled morphological phases for application as catalysts for the hydrogen evolution reaction.^97^ The general process for synthesizing these materials through FJH is illustrated in Figure 7(A). In this process, metals (M), metal oxides (MO_*x*_), metal chlorides (MCl_*x*_), or metal hydroxides (M(OH)_*x*_), among other species, are incorporated into a quartz tube with carbon black which serves a dual purpose. The conductive nature of the carbon black particles enables the Joule heating process to occur while also acting as a carbon source for the synthesis of the metal carbides. In the FJH process, the metallic species are vaporized while the carbon additives remain solid. The metal vapors react with the carbon particles to produce metal carbides, as described in the process. In comparison, the traditional process is essentially the inverse, relying on the introduction of gaseous hydrocarbons to solid metal precursors. This results in metal carbide species coated with amorphous carbon due to the additional gaseous carbon precursors. In the FJH process, the bed of precursor is connected to a capacitor bank capable of discharging large voltages in approximately 50 ms. The resulting temperature increase as a function of time after the initial voltage discharge is depicted in Figure 7(B). After the application of 100 V over a 50 ms period, the temperature increases very rapidly to ∼2700 °C, which then cools after the discharge. At these elevated temperatures, the metal precursors investigated (molybdenum chloride (MoCl_3_), tungsten oxide (WO_3_), vanadium oxide (V_2_O_5_), chromium (Cr), titanium (Ti), and boron trioxide (B_2_O_3_)) exhibit higher vapor pressures than the carbon black additives (Figure 7(C)). This enabled the synthesis of their corresponding carbides, among multiple others, with particle sizes that were roughly 10 nm −30 nm in size thus enabling their efficient use for applications in catalysis.

Similar processes have also been employed for the synthesis and processing of ceramic materials. Hu et al. employed Joule heating for the synthesis of ceramic bodies from salt and oxide precursors by sandwiching pressed pellets between carbon papers, which were connected to a power source.^221^ When a current (∼20 A) was applied to the carbon paper, the materials were heated to 3000 °C in ∼10 s which enabled the sintering of tantalum-doped Li_6.5_La_3_Zr_1.5_Ta_0.5_O_12_ (LLZTO) which is a garnet-type ceramic employed in solid-state electrolyte applications. In conventional syntheses, the long exposures of these garnet-type ceramics to elevated temperatures can result in the loss of lithium which deteriorates the performance of the materials. Using rapid Joule heating can greatly reduce the loss of lithium due to the increased kinetics of the densification process in comparison to the evaporation of lithium. Interestingly, this process was also extended to 3D-printed polymer-derived ceramics (silicon oxycarbide, SiOC) with complex geometries to produce macroscopic ceramic lattices which demonstrated utility as magnetic flux sensors.^221^ Additionally, Pérez-Maqueda et al. employed the FJH approach to synthesize transition metal diborides such as zirconium diboride and hafnium diboride.^222^ These ceramic materials exhibit ultrahigh thermal stability with a strong ability to maintain performance in extreme environments. Additionally, it was determined that employing the FJH approach resulted in large energy savings compared to conventional synthesis techniques, and that the experimental parameters could be tuned to alter the phase purity, microstructure and properties of the resulting diboride materials.

## Outlook and Conclusion

While Joule heating for synthesizing new materials has been established in the literature for at least a number of years, its widespread application is still very recent. Additionally, the adoption of Joule heating processes in industry faces multiple inherent challenges. A major challenge to address is the source of electricity to drive these processes. Large increases in power requirements may place strain on the electrical grid and also result in significant losses if the power must be transmitted over long distances. One of the major advantages of Joule-heated technologies is that they provide a method for directly using renewable energy sources, further decreasing the CO_2_ emissions associated with the process. However, renewable energy sources can fluctuate greatly in availability, requiring Joule-heated reactors that can adapt to changes in energy supplies through rapid start-up/shut-downs.^223,224^ Additionally, current reactors typically rely on the flow of reactants through a bed of pellet-shaped catalysts which are heated externally.^225^ Heating beds of pelletized catalysts can be challenging for multiple of the reactor designs described here, thus requiring careful design of the Joule heating reactor, or redesigning the established catalysts. Such extensive retrofitting can be challenging to adopt quickly at industrial scales. Another challenge is scaling these processes from the laboratory to practical industrial volumes. Although Joule heated processes require much smaller reactor volumes, mass transfer limitations, heating efficiencies, and energy loss from the electrical components must all be considered to apply them effectively. As such, there are numerous new avenues for research that could be fruitful for the widespread decarbonization of industrial syntheses, among other processes. For instance, there is significant room for improvement with regard to maximizing the efficiency of Joule heated reactions through understanding the fundamental mechanisms that govern the performance of those reactions. Leveraging advanced characterization and computational methods to understand the spatial and temporal control of heat within the reactor across nanoscopic to macroscopic length scales during the reaction process is necessary to further unlock to potential of Joule heated processes in the future. Specifically, this could aid in scaling Joule heated processes to industrially relevant scales and facilitate their widespread adoption throughout the chemical industry. It is also worth noting that most of the reactions described throughout this review were at atmospheric pressure, but many industrial chemical reactions, such as the production of syngas and industrial methane reforming, require very high pressures up to or exceeding 50 MPa.^226,227^ Joule heated reactors designed for pressure-bearing processes are virtually absent in the literature and must be developed for future applications.

While multiple Joule heated reactions were covered throughout this review, there are many other processes in the industry that could benefit from the adoption of Joule heating. For instance, electrified heating provides an excellent opportunity for the energy efficient destruction of waste materials. This has been demonstrated by Hu et al., who employed Joule heating to deconstruct plastic wastes for alternative fuels (described in section 3.4), as well as for the degradation of difficult to manage contaminants like per- and poly fluoroalkyl substances (PFAS). Briefly, Ali et al. have demonstrated the degradation of various PFAS through the induction heating of stainless-steel reactors loaded with PFAS molecules.^228^ This technique was employed to rapidly degrade PFAS in the solid state, indicating its applicability to spent sorbents that have been used to adsorb PFAS from wastewater sources that provide more efficient management of these harmful contaminants. Another unique advantage that Joule heating confers is the ability to fuse heating elements in order to form continuous networks and/or composite materials. For example, carbon nanofibers were welded together into a three-dimensional interconnected matrix through Joule heating up to 2200 °C.^229^ This process resulted in ultrafast graphitization that increased bulk electrical conductivity by 4 orders of magnitude (380 S/cm). With a similar electrothermal shock approach, Fu et al. demonstrated a cross-scale manufacturing approach to prepare mechanically robust composites containing nanoscale (carbon nanotubes) and macroscale (glass fiber) components that were welded together during rapid Joule heating.^230^ These nanowelding strategies demonstrate how Joule heating processes can be utilized in composite manufacturing as well as in material repair to extend service life.^231^

In addition to the application of Joule heating to other industrial processes, significant amounts of work need to be dedicated to optimizing and scaling existing technologies to meet industrial demands. This can be accomplished through further material design to maximize the temperature outputs with reduced power demands, in addition to the comprehensive examination of the impacts of varied flow fields on reactant conversion which can be extended to the production of tailored reactor designs. Additionally, as renewable energy sources become increasingly prevalent, their incorporation into Joule-heated processes and their impact on technoeconomic analyses and lifecycle assessments must be investigated. Altogether, Joule heated syntheses can be a very promising approach to increasing the efficiency of existing industrial processes, as well as for the large-scale production of novel materials, and there are many exciting research opportunities within this area.
